# Lysophosphatidylcholine as an adjuvant for lentiviral vector mediated gene transfer to airway epithelium: effect of acyl chain length

**DOI:** 10.1186/1465-9921-11-84

**Published:** 2010-06-23

**Authors:** Patricia Cmielewski, Don S Anson, David W Parsons

**Affiliations:** 1 Dept of Respiratory and Sleep Medicine, Women's and Children's Hospital, 72 King William Rd, North Adelaide, SA, 5006, Australia; 2 Gene Technology Unit, SA Pathology, Women's and Children's Hospital, 72 King William Rd, North Adelaide, SA, 5006, Australia; 3 Department of Paediatrics, University of Adelaide, Adelaide, SA, 5005, Australia; 4 Centre for Stem Cell Research, University of Adelaide, Adelaide, SA, 5005, Australia; 5 Women's and Children's Research Health Institute Inc, 72 King William Rd, North Adelaide, SA, 5006, Australia

## Abstract

**Background:**

Poor gene transfer efficiency has been a major problem in developing an effective gene therapy for cystic fibrosis (CF) airway disease. Lysophosphatidylcholine (LPC), a natural airway surfactant, can enhance viral gene transfer in animal models. We examined the electrophysiological and physical effect of airway pre-treatment with variants of LPC on lentiviral (LV) vector gene transfer efficiency in murine nasal airways *in vivo*.

**Methods:**

Gene transfer was assessed after 1 week following nasal instillations of a VSV-G pseudotype LV vector pre-treated with a low and high dose of LPC variants. The electrophysiological effects of a range of LPC variants were assessed by nasal transepithelial potential difference measurements (TPD) to determine tight junction permeability. Any physical changes to the epithelium from administration of the LPC variants were noted by histological methods in airway tissue harvested after 1 hour.

**Results:**

Gene transduction was significantly greater compared to control (PBS) for our standard LPC (palmitoyl/stearoyl mixture) treatment and for the majority of the other LPC variants with longer acyl chain lengths. The LPC variant heptadecanoyl also produced significantly greater LV gene transfer compared to our standard LPC mixture. LV gene transfer and the transepithelial depolarization produced by the 0.1% LPC variants at 1 hour were strongly correlated (r^2 ^= 0.94), but at the 1% concentration the correlation was less strong (r^2 ^= 0.59). LPC variants that displayed minor to moderate levels of disruption to the airway epithelium were clearly associated with higher LV gene transfer.

**Conclusions:**

These findings show the LPC variants effect on airway barrier function and their correlation to the effectiveness of gene expression. The enhanced expression produced by a number of LPC variants should provide new options for preclinical development of efficient airway gene transfer techniques.

## Introduction

The search for a safe and effective gene therapy for cystic fibrosis (CF) airway disease has been underway for more than 15 years, and throughout this time three issues have underscored the slow progress in this field: the poor efficiency of gene transfer; the short persistence of gene expression; and the abrogation of initial gene expression by host inflammatory and immune responses [1,2].

Poor efficiency can be seen as a problem in general for gene transfer to airways - evolution has produced a extremely effective series of protective barriers and that efficiently block, remove or destroy the bulk of vector doses delivered into the airway [3,4]. For a genetic disease like CF, highly-persistent gene correction or complementation is necessary to counter the life-long effects of the disease; in this regard lentivirus vectors are an obvious choice because of their longevity of expression and depending on their pseudotype, their ability to transduce many cell types. Inflammatory and immune responses have limited the therapeutic effectiveness of many other viral vectors such as adenovirus and adeno-associated adenovirus [5-7]. However, helper-dependent adenovirus have shown enhanced efficiency and surprisingly high levels of expression in mouse [8], rabbit and baboon lungs [9,10] when delivered in conjunction with lysophosphatidylcholine (LPC). Nevertheless, the long term persistence of expression from these vectors has not been adequately assessed. We have also demonstrated that high level long-lasting lentiviral gene expression can be produced in intact nasal airways of normal and CF mice after LPC pre-treatment [11,12].

The major action of LPC in all these circumstance appears to be the improvement of vector access to appropriate airway cell surface receptors as well as transient disturbance of epithelial surface barrier function to permit vector particle access to basolateral receptors and to deeper-lying basal cells that may have progenitor-like qualities. Following the success of our pre-treatment studies using a standard and readily available natural form of LPC (derived from egg yolk, a mixture of palmitoyl/stearoyl forms), we report here on studies designed to determine whether molecular variants of LPC could produce more effective enhancement of LV gene transfer and/or reduce the potential for damage to the airway epithelium.

## Materials and methods

### Nasal Dosing

Studies were conducted with approval from the Animal Ethics Committee at the Women's and Children's Hospital, Adelaide, SA. Female C57Bl/6 mice, 8-10 weeks of age were anaesthetised with 10 μl/g body weight of Domitor (0.1 mg/ml, Orion Corporation, Finland) and Ketamine (7.6 mg/ml, Parnell Laboratories Aust Pty Ltd) mixture, delivered i.p. Anaesthesia was reversed with 2 μl/g i.p. injection of atipamazole (0.5 mg/ml, Orion Corporation, Finland).

LPC variants were obtained from Sigma-Aldrich Chemical Co: L4129 egg yolk (the standard LPC molecule used previously), L3135 decanoyl, L5629 lauroyl, L6629 myristoyl, L5254 palmitoyl, L5257 heptadecanoyl, L2131 stearoyl, and L1881 oleoyl. LPC variants differed only in the length of their acyl chain (Table 1). PBS was used as a vehicle-control and in preparation of both the 0.1% and 1% concentrations.

**Table 1:** Structural formula of the LPC variants, defined by the lengths of their acyl chains.

LPC	Acyl Chain	Structure	**M. W**.
Decanoyl	C10:0	C_18_H_38_NO_7_P	412
Lauroyl	C12:0	C_20_H_42_NO_7_P	439
Myristoyl	C14:0	C_22_H_46_NO_7_P	468
Palmitoyl	C16:0	C_24_H_50_NO_7_P	496
Heptadecanoyl	C17:0	C_25_H_52_NO_7_P	510
Palmitoyl:Stearoyl	C16:C18	66%:33%	505
Stearoyl	C18:0	C_26_H_54_NO_7_P	524
Oleoyl	C18:1	C_26_H_52_NO_7_P	522

#### Gene Transfer

Instillations were made into the right nostril *via *a micropipettor with a gel-loading tip (Finnpipette), as described previously [11,13]. Pre-treatment with LPC variant or control (PBS) was *via *a 4 μl aliquot one hour prior to a 20 μl bolus of the lentivirus (LVLacZ) vector (self-inactivating HIV-1, VSV-G pseudotype under transcriptional control of the simian virus 40 early promoter), at a titre of 4 × 10^7 ^tu/ml, the latter delivered in 2 × 10 μl aliquots over a minute, *via *passive (inhalation-driven) fluid uptake [11].

#### Assessment of gene expression

**Figure 1:**
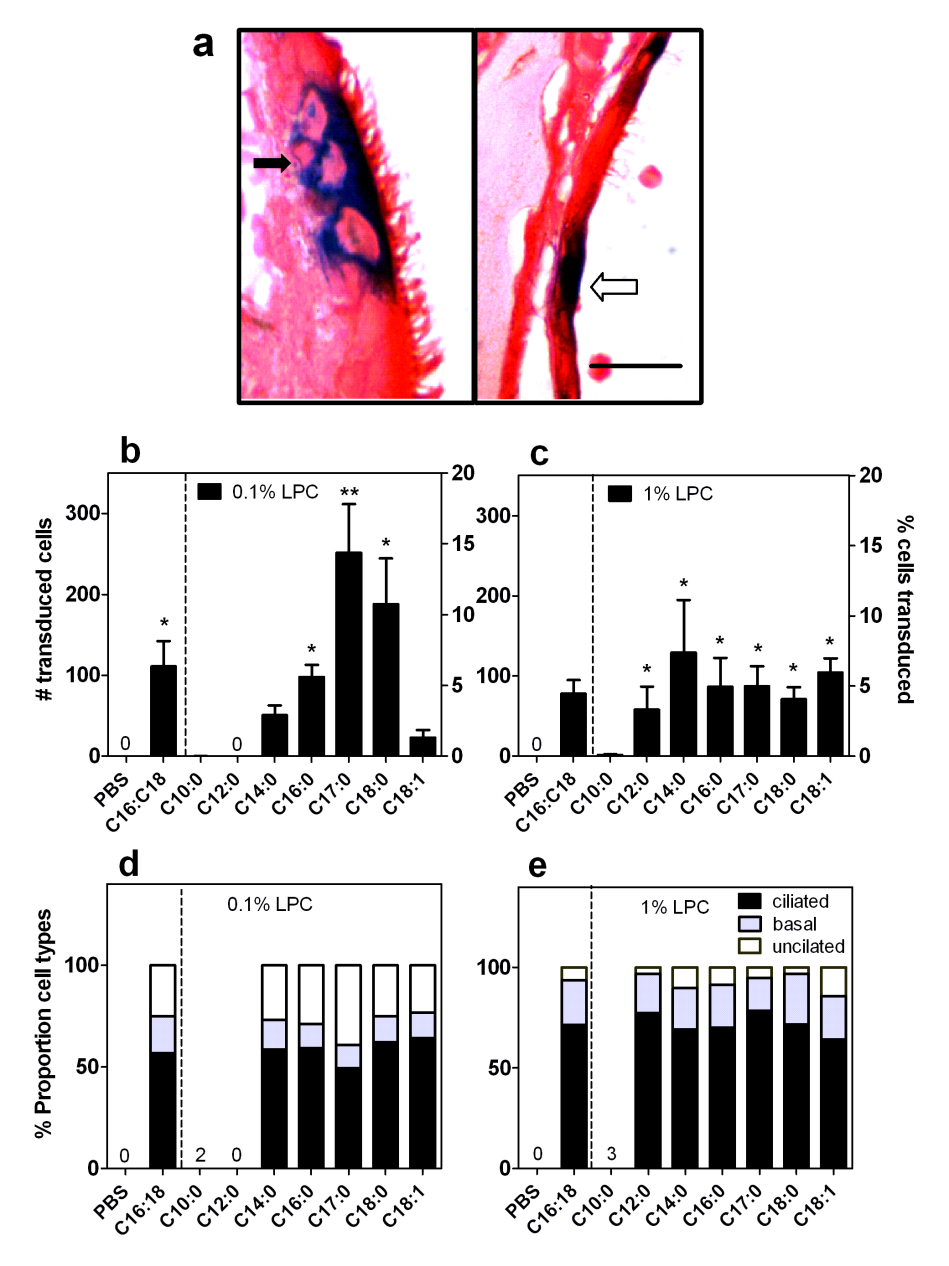
**LV gene transfer in nasal respiratory epithelium**. (a) Histological examples of LV transduced (blue staining) cell types after 1 week. Left panel shows transduced ciliated and basal cells (solid arrow) and unciliated cells (open arrow, right panel). Safranin O staining, scale bar = 10 μm. LV Gene transfer at 1 week following 0.1% (b) and 1% (c) LPC variant pre-treatment. *p < 0.05 ANOVA against PBS, **p < 0.05 t-test C17:0 vs standard LPC (C16:C18), (n = 6/group). The proportion of respiratory cells types transduced following 0.1% (d) and 1% (e) LPC variant pre-treatment. Note: PBS is the same group of control animals in b-e) for Figure 1; a, b) for Figures 3, 5 and 7; and a-d) for Figure 4.

Mice were sacrificed 7 days post-treatment to quantify gene transfer. Heads were processed to reveal LacZ gene expression *via *the standard X-gal method previously described [11,12]. The numbers and percentages of positive LacZ cells were counted in respiratory and transitional epithelium in three standard head cross sections, detailed in [12]. Sections were stained with haematoxylin and eosin (H&E) to reveal cellular identity and morphology, or safranin-O to provide a high contrast counterstain for assessment of β-galactosidase gene expression (as blue-stained cells, Fig 1a.).

### Electrophysiological and Histological Actions of LPC

#### Epithelial Potential Difference

For assessment of tight junction permeability, the electrophysiological integrity of mouse nasal airways was measured using the transepithelial potential difference (TPD) measurement as previously described [11]. Briefly, fine polyethylene tubing was inserted ~2.5 - 3 mm into the right (dosed) nostril of the anaesthetized mouse, and the TPD was recorded using DataStudio (Version 1.9.8r2, Pasco Scientific, USA) until a steady plateau of at least 1 min was obtained. The ΔPD was calculated by subtracting the TPD value recorded under basal conditions (perfusion with Krebs-buffered Ringers) from the value recorded using low-chloride conditions (Krebs with NaCl replaced with Na gluconate). The low chloride setting was used to provide a large TPD value upon which any changes induced by LPC could be more readily detected. A large span of TPD value permits the readout variability to be a smaller proportion of the signal, decreasing the experimental-animal samples sizes required. Once baseline ΔPD was established a 4 μl bolus of either PBS or LPC variant was instilled into the same nostril and the change in low chloride TPD measurement was continuously recorded for a further hour. This provided an effect time-course and a final value for the LPC-induced change that would be present in our gene transfer studies, since the subsequent gene vector deliveries were performed 1 hour after LPC treatment.

#### Histology

Two weeks after the initial treatment the same LPC treatment for that mouse was repeated into the right nostril. Animals were sacrificed via CO_2 _asphyxiation after 1 hour, the usual time of our gene vector administration. Heads were processed for histological examination to produce standard cross sections (noted above, and see [12]) and stained with H&E or Alcian Blue/Periodic Acid Schiff reagent, to identify effects on epithelial morphology and goblet cell mucin presence respectively. Goblet cell numbers were counted in the first two standard cross sections (equivalent to levels 6 and 16 in Mery et al [14], in which the nasal septum separates both nostrils) in both the left and right nostrils in all animals. The percentage of goblet cells in the treated vs untreated side was then calculated. A rank scale based on the level of epithelial disturbance in the treated nostril was used to compare to the disturbance in the untreated nostril. Both treated and untreated regions could be examined in the same sections. The scale used was: 0 = no difference, 1 = Loss of goblet mucin, 2 = loss of some cilia, 3 = physical disruption of cell-cell junctions, 4 = areas of exfoliation in the epithelial layer.

### Statistics

All data are expressed as the mean ± standard error of the mean, and statistical analysis was performed using SigmaStat 3.01 (SPSS, Chicago, IL). Multiple treatment groups were analyzed by one-way analysis of variance (ANOVA) using post-test multiple comparison techniques with statistical significance set at *p *= 0.05, and power = 0.8. Student t-tests were performed to compare 2 groups, and standard transformations or appropriate nonparametric methods were utilized where data did not satisfy normality assumptions.

## Results

### LV Gene Expression

We first examined the effect of pre-treatment of a range of LPC variants at a low (0.1% w/v) and high (1% w/v) concentration on LV mediated gene transfer measured 1 week later (Fig. 1). The biologically occurring LPC molecules palmitoyl-C16:0, stearoyl-C18:0 and oleoyl-C18:1 are phospholipids found in the fatty acids of many mammalian tissues [15-17]. Decanoyl-C10:0, lauroyl-C12:0 and myristoyl-C14:0 are LPC variants with short acyl chains and heptadecanoyl-C17:0 has an un-even acyl chain length that is not found in nature. We tested these LPC variants against a PBS control as well as our standard LPC (egg yolk derived LPC, a mixture of palmitoyl and stearoyl (C16:C18) forms).

Gene transduction was significantly greater compared to control (pre-treatment with PBS) at the low concentration for our standard LPC and 3 of the new LPC variants, palmitoyl-C16:0, heptadecanoyl-C17:0 and stearoyl-C18:0 (Fig. 1b, p < 0.05, ANOVA). The LPC variant heptadecanoyl-C17:0 also produced significantly greater LV gene transfer compared to our standard LPC mixture (p < 0.05, t-test). However, for the high dose tested (1%) all LPC variants, except decanoyl-C10:0, produced LV gene transfer greater than control (p < 0.05, ANOVA) and were equivalent to our standard palmitoyl/stearoyl LPC mixture (Fig. 1c).

Ciliated, basal and unciliated cells were the only respiratory cell types transduced (Fig. 1 a, d-e). The proportion of these three cell types differed with the different concentration of LPC but not with the variants of LPC pre-treatment. For 0.1% LPC concentration the majority of cells transduced were ciliated (60%), 26% were unciliated and basal cells compromised approximately 14% (Fig. 1d). At the higher concentration of 1% LPC ciliated cells were still the majority transduced (72%) however the proportion of basal cells was 21% with unciliated cells at 7% (Fig. 1e). No olfactory, goblet or squamous epithelial cells were transduced.

### Electrophysiological Action of LPC

**Figure 2:**
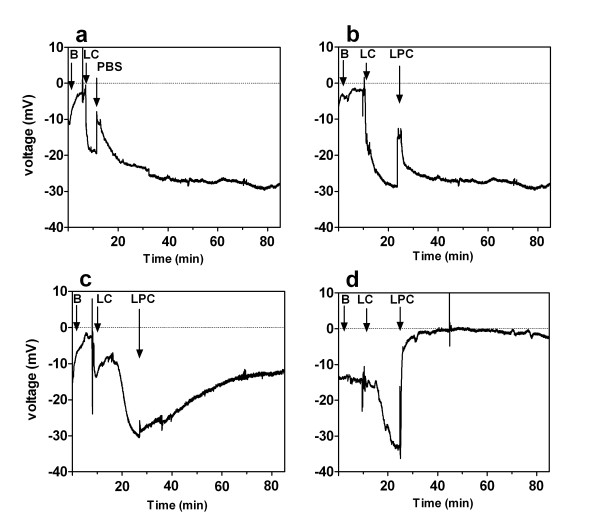
**Representative traces of nasal potential difference measurements**. Examples of TPD traces, monitored for up to 60 mins after addition of (a) PBS, (b) 0.1% LPC-decanoyl, (c) 0.1% LPC-standard and (d) 0.1% LPC-heptadecanoyl. B = basal Krebs, LC = low-chloride Krebs, LPC= addition of 4 μl bolus of LPC into same nostril, PBS= addition of 4 μl bolus of PBS into same nostril. Traces are each representative of n = 5-6 mice.

From the above experiments we selected four LPC variants that displayed key differences in LV gene transduction responses at both concentrations for further study. Pre-treatment with decanoyl-C10:0 displayed no gene transfer, stearoyl-C18:0 and heptadecanoyl-C17:0 had similar or greater gene expression than our standard LPC, and the fourth variant oleoyl-C18:1 produced an intermediate response, at the two concentrations tested. These LPC variants, our standard LPC mixture, and a PBS control were examined for their effect on epithelial membrane physiology. The assessment of epithelium electrical potential difference (a measure of electrophysiological integrity in part based on tight junction permeability) was *via *the nasal transepithelial potential difference (TPD) measurement. Under low-chloride perfusion (see methods) a 4 μl aliquot of a particular LPC variant was instilled and the TPD recorded continuously for a further 1 hour; four examples are shown in Fig. 2.

**Figure 3:**
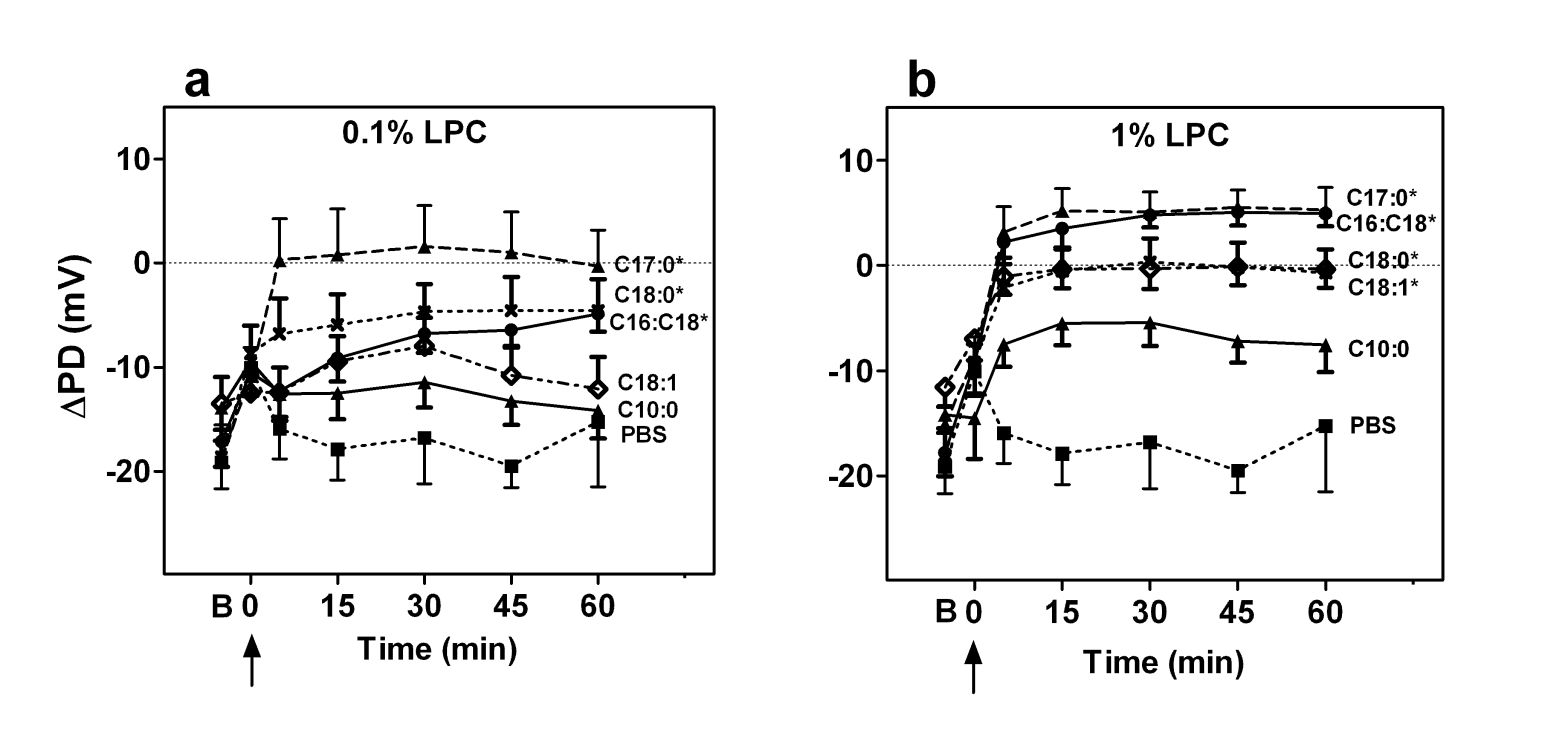
**Summary of change in potential difference after LPC administration**. ΔPD monitored for 60 mins post (a) 0.1% and (b) 1% LPC variant instillation. B designates baseline ΔPD. Arrow designates instillation of LPC variant (or PBS); *p < 0.05, ANOVA compared to baseline (n = 8/group).

The PBS control (Fig. 2a) and the LPC variants that resulted in no or little gene transfer (i.e. decanoyl-C10:0 (Fig. 2b) and 0.1% oleoyl-C18:1) produced a brief depolarization in the TPD level when the 4 μ1 bolus was instilled but had returned to pre-instillation (baseline) levels by the 1 hour time point. The LPC variants that produced strong LV gene transfer showed either a gradual (Fig. 2c) or an immediate depolarization towards zero that persisted for the entire 1 hour post-instillation period (Fig. 2d). All LPC variants that displayed a significant difference in mouse nasal ΔPD at the 1 hour time point compared to the baseline ΔPD (Fig. 3a &3b, p < 0.05, ANOVA) corresponded to those mice displaying more efficient LV gene transfer at 1 week.

**Figure 4:**
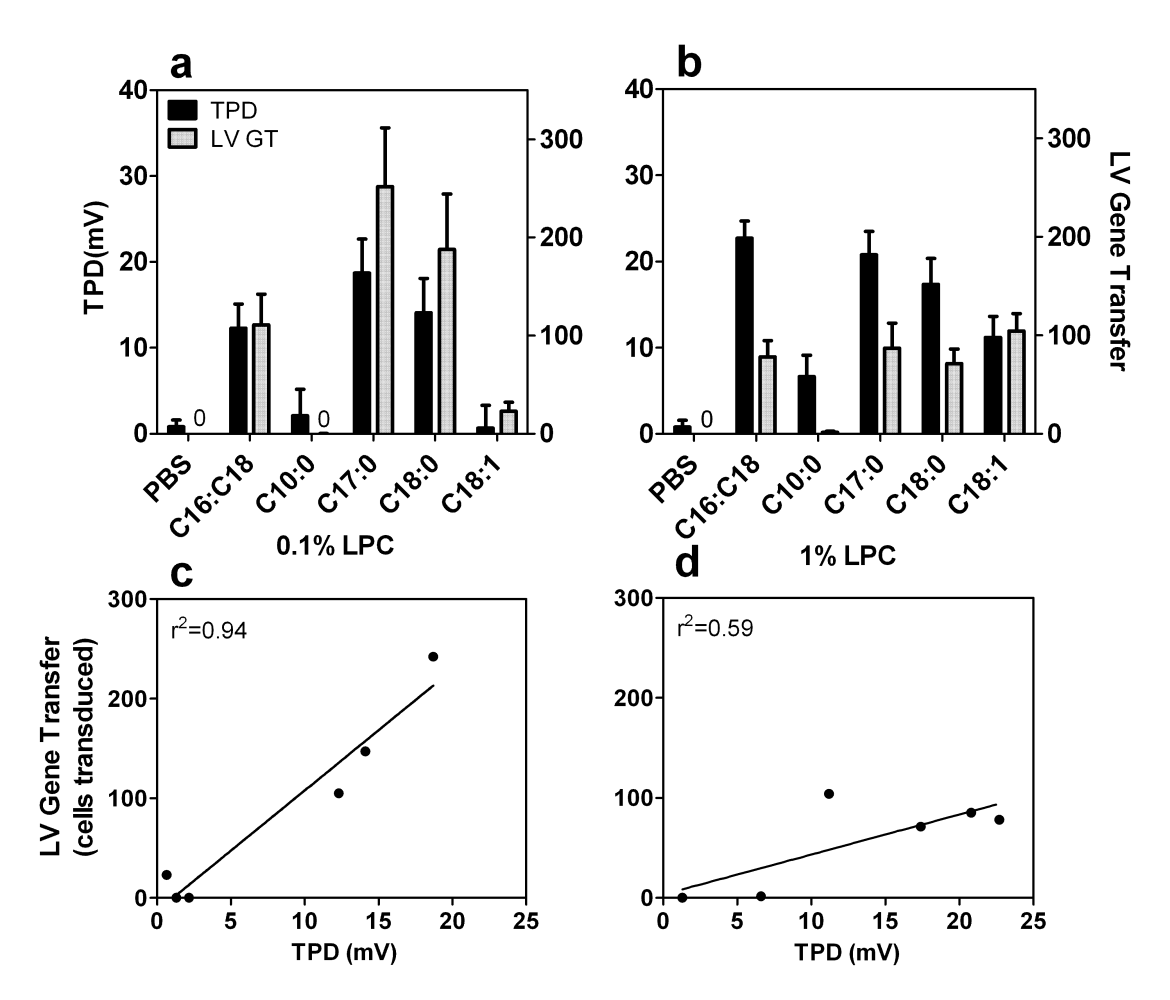
**Correlation between LV gene transfer and 1 hr TPD depolarization**. Change in low-chloride (see Methods) TPD at 60 min post LPC (solid bars) compared to LV gene transfer (grey bars) and corresponding correlation graph. a, c) 0.1% LPC and b, d) 1% LPC variants (n = 6-8/group).

More specifically, at 1 hour post-instillation, i.e. at the usual time of gene vector instillation, the higher depolarizations in low-chloride TPD were correlated with higher LV gene expression. LV gene transfer and the TPD depolarization produced by the 0.1% LPC variants were strongly correlated (r^2 ^= 0.94 Pearson's correlation, p = 0.001, Fig 4a, c), but at the 1% LPC concentration the correlation was less strong (r^2 ^= 0.59, p = 0.077, Fig. 4b, d).

### Epithelial Disturbance of LPC

**Figure 5:**
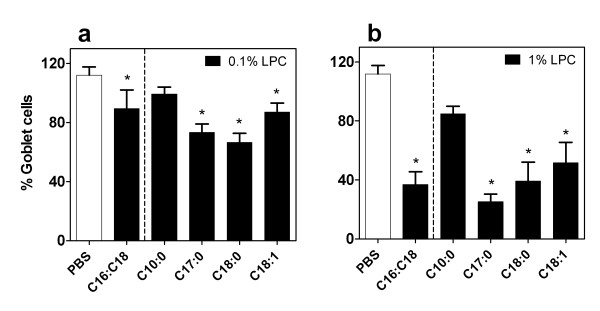
**Mucin release after administration of LPC variants**. Percentage of goblets cells remaining in nasal epithelium of treated vs untreated side, at 1 hr post a) 0.1% LPC and b) 1% LPC administration. *p < 0.05, ANOVA vs PBS (Holm-Sidak method, n = 8/group).

Two weeks after TPD measurement (a time sufficient for full regeneration of epithelium damaged by these procedures [18]), the same LPC variant type and dose delivered to that mouse was again instilled into the right nostril. One hour later mice were sacrificed for histological preparation of the nasal airways and assessed (compared to the untreated nostril epithelium [19]) for changes in goblet cell number and mucin presence. In the untreated nostril an average of 260 goblet cells were counted per mouse. Any physical changes to the integrity of the epithelial layer on the treated side were also noted. There was a significant reduction in the number of mucin-containing goblet cells of the epithelial layer in response to nasal instillation after all LPC variants except for decanoyl-C10:0 and when the PBS control treatment was employed (Fig. 5a-b, p < 0.05, ANOVA, Holm-Sidak).

**Figure 6:**
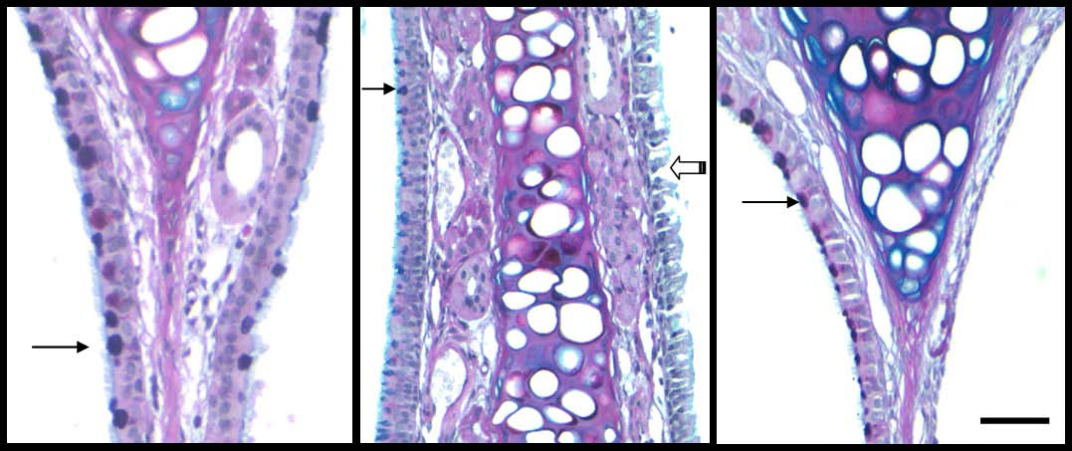
**Nasal respiratory epithelia following LPC instillation**. Examples of epithelial disturbance after LPC administration at the 60 min time point. Left side of tissue section is untreated and right side is treated. Goblet cells are those cells containing the compact darkly-stained contents (examples shown at dark arrows, untreated side). Scale bar = 50 μm. Low level effects, ranked at Level 1 (see Methods), displayed some loss of goblet cell mucins when compared to the untreated side (e.g. left panel). Disruption of the cell membrane junctions between cells, loss of some cilia and reduction in goblet mucin was ranked as Level 3 (e.g. middle panel, open arrow, treated side). An example of a region of complete exfoliation of epithelial layer produced by 1% LPC instillation is shown for comparison (Level 4, right panel).

**Figure 7:**
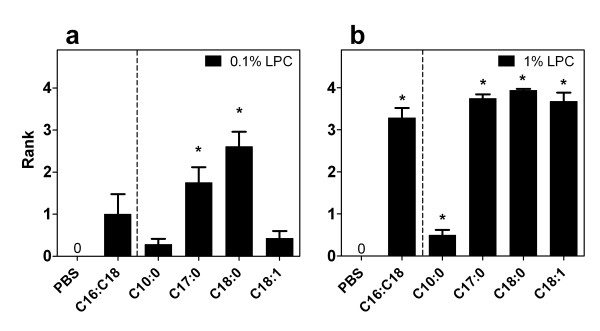
**Rank measurements of epithelial disruption after LPC variant administration**. Epithelial disturbance due to a) 0.1% and b) 1% LPC administration, at the 60 min time point. *p < 0.05, Wilcoxon Signed Rank vs PBS (n = 8/group).

As a surfactant with detergent properties, LPC given at high concentrations has the potential to produce physical disturbance of the epithelium. We ranked the effects of LPC on the epithelium ranging from no effect; through to mild perturbations, loss of cilia, and physical disruption of cell-cell junctions; to areas of epithelial cell shrinkage and/or exfoliation of the epithelial layer (Fig. 6). PBS control treatments as well as those LPC variants ineffective at sustaining a TPD reduction (decanoyl-C10:0, 0.1% oleoyl-C18:1) produced no physical disruption of the epithelial layer in the treated side compared to the untreated side. However, the LPC variants that displayed minor-moderate levels of disruption to this layer (ranging from loss of cilia to disruption of cell-cell membranes) were clearly associated with higher LV gene transfer (Fig 7a, p < 0.05, ANOVA). Indeed, even the LPC variants that produced areas of cellular exfoliation (Fig. 7b, p < 0.05, ANOVA) could nevertheless enhance LV gene transfer, albeit at a lower efficacy.

In all studies procedures were well tolerated, post procedure weight changes were unremarkable and there was no related morbidity and mortality.

## Discussion

Despite the strong interest in gene therapy as a cure or treatment for the chronic and progressive disease that overtakes the cystic fibrosis lung, no clinically therapeutic benefits have yet appeared due largely to ineffective vector transduction [20] and host immune and inflammatory responses [5,6]. If the traditionally poor efficiency of gene transfer can be improved there is the potential to produce substantial and positive gene expression outcomes. Increasing the efficiency of gene transduction would permit a reduction of the dose of vector and its contaminants that should automatically help to reduce the intensity of the immune and inflammatory responses. The use of an enhancer such as LPC provides an effective method to reduce the vector dose necessary to achieve a given level of gene expression *in vivo*.

The efficiency of gene transfer with any vector can be improved by increasing the ability of vector particles to reach and remain near their target cells [21]. In normal circumstances the availability of the majority of vector particles delivered to the airways for transduction process are rapidly and substantially reduced by the natural barriers to airway infection. These include the mucus layer, the mucociliary clearance that transports particles out of the airway [22,23] and the glycocalyx [24] that can bind vector particles that escape these initial barriers and all can prevent vector contact with the relevant cell-membrane receptors. In terms of vectors that can also target basolateral receptors, such as VSV-G pseudotyped LV vectors, the tight junctions between the cells provide an additional barrier to transduction that must be considered. Although airway gene transfer using a simian LV vector pseudotyped with a Sendai virus envelope [25] or a feline LV vector pseudotyped with a baculovirus GP64 envelope [26] can effectively target the apical receptors in murine nasal airway, a large dose (volume and titre) of the vector and long residence time was needed to overcome these initial barriers. The combination of LPC pretreatment with the VSV-G pseudotyped LV vector permits transduction at apical as well as basolateral sites, providing immediate (apical) and potentially long term transduction via basally located progenitor cells [27].

Since the structural changes present in the molecular variants of LPC are likely to alter their biological function or effectiveness, we examined the effect of different molecular variants of LPC on their ability to enhance LV gene transfer. We used LPC variants based on their differing acyl chain length - from C10:0 decanoyl to C18:1 oleoyl- as well as at a low (0.1%) or a high (1%) concentration. Most biologically occurring LPC molecules possess longer acyl chain lengths, between C16:0 and C22:6 [17,28].

The lack of effectiveness with decanoyl-C10:0 and other shorter acyl chain LPC molecules mirrors the known lack of effects on cell toxicity [29] and membrane permeability [30] noted elsewhere. At the 0.1% concentration LV gene expression was absent or not significantly different to control (PBS) for the decanoyl-C10:0, lauroyl-C12:0, myristoyl-C14:0 and oleoyl-C18:1 LPC pre-treatment. In contrast, pre-treatment with palmitoyl-C16:0 or stearoyl-C18:0 produced similar levels of gene transfer to our standard LPC mixture (containing both C16:0 and C18:0). Heptadecanoyl-C17:0 pre-treatment produced the strongest LV gene expression of all those tested at the 0.1% concentration. These findings are consistent with those in other studies where 0.1% LPC has produced successful airway gene transfer in mice, rabbits and baboons [8-10]. This present study also demonstrates that LPC pretreatment can be effective at 1/10^th ^of the dose previously used in mouse airway to enhance LV gene expression [12]. At the high concentration (1%) every LPC variant except decanoyl-C10:0 produced similar levels of LV gene transfer compared to our standard LPC mixture.

The percentage of gene transduction ranged from 1.5% (oleoyl-C18:1) to 15% (heptadecanoyl-C17:0) of the epithelial respiratory cell layer, and if applicable to CFTR gene transfer maybe sufficient for effective correction of the CF gene defect in airways [31,32]. The majority of respiratory cell types transduced at either concentration were ciliated cells (60-72%), with the only other cell types transduced being unciliated and basal cells. There was a modest increase in the proportion of basal cells compared to unciliated cells after 1% LPC pre-treatment. The increase in basal cell transduction could provide a basis for effective long term LV expression, as this is the niche where progenitor-like stem cells are thought to reside [27].

We sought explanations for these differences in gene transfer via electrophysiological and histological analyses. The magnitude of depolarization of the electrical response (TPD) one hour after 0.1% LPC administration (the time of vector dosing) was strongly correlated with higher gene transfer, as measured by counts of cells expressing LacZ (Fig 4a, c). Since the TPD is reduced as tight junction barrier function is lost [18] this finding supports the idea that tight junctions become permeabilised by LPC after treatment with most of the variants. Such permeabilisation is known to permit viral vector particles to access the relevant basolateral receptors [33], with the ensuing increase in vector particle binding to appropriate basolateral receptors resulting in increased airway gene transfer. Conversely, when little or no depolarization was present 1 hour after the LPC treatment, poor gene expression was observed (Fig. 4: see decanoyl-C10:0 treatment group).

Another example of the influence of epithelial barrier permeability was with 0.1% oleoyl-C18:1 LPC. Here the change in potential difference was brief and transient, with the TPD having returned to baseline by the usual time of vector instillation, and the level of gene transfer produced was low. This relationship between the TPD and LPC pre-treatment suggests that improved gene transduction may have been possible had the time of vector instillation corresponded to the maximum depolarization of TPD (e.g. 30 min for this oleoyl LPC variant). Optimization of successful gene transfer for a particular LPC variant may depend on selecting the best pre-treatment timing and LPC concentration for that variant, and future studies could determine if the shorter timing intervals are more effective for variants such as oleoyl. This notion is further supported by our finding that at the high 1% LPC concentration only the decanoyl-C10:0 variant remained unable to induce necessary membrane permeability as measured electrophysiologically, and this was consistent with the absence of gene transfer.

At the two concentrations tested, the LPC variants displayed differences in the degree of effect on the airway epithelium morphology. We examined both the release of cell-bound mucosubstances and changes to the physical integrity of the epithelium. Mucosubstance changes were particularly evident as a loss of mucin granules from epithelial goblet cells; this is a normal airway response in defense against foreign stimuli and particles and will aid efficient mucus based particle capture and subsequent mucociliary clearance. The lack of effect from control vehicle administrations at the one hour time point (Fig. 5) showed there was no significant release of mucin from the epithelial cells from the delivery process of PBS, and this was also observed after decanoyl-C10:0 administrations. Consequently, the LV vector that is instilled at this time will still encounter a primed mucociliary clearance system (as well as intact tight-junctions), and this may explain in part, the absence of any enhancement of gene transfer by PBS or decanoyl-C10:0 LPC when used as a pretreatment agent. The significant increase in the release of mucin from the epithelial goblet cells in reaction to the remaining LPC variants suggests an innate mucin-based defense exists towards these molecules. Thus, at the time of gene vector instillation most goblet cells will have previously released their mucin. We speculate that the initial loss of mucins in response to LPC delivery may be an additional factor in increasing gene transfer and expression, by leaving the airway temporarily unable to capture and remove the vector gene particles when they are delivered 1 hour later.

All LPC pretreatments that produced effective gene expression displayed physical perturbations of the epithelial layer apparent 1 hour after LPC administration. These included loss of cilia, disruption of cell-cell junctions and even exfoliation of some areas (Fig. 7). Where cellular exfoliation was induced in some regions (typically using the 1% doses of LPC variants) we found that gene expression decreased (significantly for heptadecanoyl-C17:0 and stearoyl-C18:0 only) compared to that produced by the low (0.1%) concentration of LPC. The high LPC concentration produced greater physical disruption than the low LPC concentration, but the level of gene transfer was similar in majority of cases. If the effect of the high dose LPC is mainly to damage the epithelium, the potential for production of gene expression maybe lost or reduced because of cell re-growth [34]. That is, as much gene expression may be lost, as would be regained, by creation and transduction of newly formed epithelial cells. However, these physical disturbances to the epithelial layer were in all cases (even at 1%) temporary, because the gene expression noted at 1 week was within an intact epithelial layer. Our data regarding the level of gene transfer achieved, the effect on mucin release, changes in electrophysiology, and epithelial disturbance supports our selection (and that of others [8,9]) to use a low dose (0.1%) of LPC for pre-treatment or co-instillation in the production of enhanced LV gene expression [12]. In addition, the widely used C16:C18 mixture remains a good choice of LPC variant. The use of LPC pretreatment also allows for lower volumes and titres of a LV vector to achieve effective gene expression and therefore has the potential to reduce immune responses. However, heptadecanoyl-C17:0 used at the 0.1% concentration (Fig. 1b) can provide significant improvement in gene transfer effectiveness over all other LPC variants tested here. For that reason this LPC variant may be worthy of further investigation, but some caution may be required for *in vivo *application and/or clinical development as this molecule is an entirely synthetic species not normally present in biological systems.

In summary, optimization of the LPC species, the concentration, and the timing of the dose prior to vector delivery can identify potentially valuable improvements in gene expression efficiency in this *in vivo *gene transfer enhancement setting. The potential advantage in a reduction of immune responses to the transgene of interest may add to the benefits of the pre-treatment enhancement itself. For successful gene therapy for diseases such as cystic fibrosis, arranging significant improvements in gene expression efficacy represents a valuable step in the development of techniques suitable for use in airway gene transfer clinical trials.

## Conclusions

We have shown that LV gene transduction in murine airways can be significantly enhanced by the optimization and selection of LPC species as a pre-treatment regime. The effect of LPC variants on airway barrier function strongly correlated with gene transduction efficiency, providing further development towards pre-clinical gene therapy protocols.

## Abbreviations

CF: cystic fibrosis; H&E: haematoxylin and eosin; LPC: lysophosphatidylcholine; LV: lentiviral; PBS: Phosphate buffered saline; TPD: transepithelial potential difference.

## Competing interests

The authors declare that they have no competing interests.

## Authors' contributions

DSA and DWP were both group leaders for these experiments and contributed to study design. PC performed all animal experiments, collected data, carried out laboratory, histological and statistical analyses and drafted the manuscript. All authors read and approved the final manuscript.
